# Karyotype characteristics, chromosomal polymorphism and gene COI sequences of *Chironomus heteropilicornis* Wülker, 1996 (Diptera, Chironomidae) from the South Caucasus

**DOI:** 10.3897/CompCytogen.v13i4.35572

**Published:** 2019-10-31

**Authors:** Mukhamed Kh. Karmokov

**Affiliations:** 1 Tembotov Institute of Ecology of Mountain territories RAS,I. Armand str., 37a, Nalchik 360051, Russia Tembotov Institute of Ecology of Mountain territories, Russian Academy of Sciences Nalchik Russia

**Keywords:** Diptera, Chironomidae, *Chironomus heteropilicornis*, polytene chromosomes, chromosome polymorphism, South Caucasus.

## Abstract

The study presents data on the karyotype characteristics, features of chromosomal polymorphism and the gene COI sequences of *Chironomus heteropilicornis* Wülker, 1996 (Diptera, Chironomidae) from the South Caucasus. We found 8 banding sequences in the Caucasian population. Overall, The Caucasian population of the species can be characterized as having a low level of polymorphism. We found one new banding sequence hpiA2 in the banding sequence pool of *Ch. heteropilicornis*. We observed inversion polymorphism only in the arm F. The dendrogram of genetic distances by Nei criteria (1972) shows a clear separation of the Caucasian population from populations of Siberia. At the same time, the distance between populations of Siberia and the population of South Caucasus (0.379–0.445) almost reach the mean distance (0.474 ± 0.314) between subspecies (Gunderina 2001). Due to this, we can assume that the population of South Caucasus separated from Siberian populations at the level of subspecies. Constructed on data for COI gene sequences the phylogenetic tree estimated by the Bayesian inference shows that the sequences of *Ch. heteropilicornis* from the South Caucasus form a separate line in the general branch of *Ch. heteropilicornis* sequences. At the same time, calculated K2P genetic distances between *Ch. heteropilicornis* sequences from Norway and Caucasus (2.0–2.2%) do not exceed the 3% threshold for the genus *Chironomus*.

## Introduction

Wolgang F. Wülker first described *Chironomus heteropilicornis* Wülker, 1996 from Sweden and Finland. According to the Fauna Europaea web source (Pape and Beuk 2016) the species is known in Europe from Sweden and Finland. However, according to Kiknadze and Istomina (2011) and Kiknadze et al. (2016), the species was also found in North Germany (Aldorf-Markonah, about 40 km south-west of Bremen) and Russia (several sites from the Republic of Sakha (Yakutia)).

The species *Ch. heteropilicornis* belongs to *Ch. pilicornis* group of closely related species. The group was proposed by Wülker (1996) and consists of two species: *Ch. heteropilicornis* and *Ch. pilicornis* Fabricius, 1787.

In the first description of karyotype of *Ch. heteropilicornis*Wülker (1996) presented mapping of arms A, C, D, E, and F made according to mapping system created by Keyl (1962) and Dévai et al. (1989). The description of chromosomal polymorphism was also presented in that study. Almost simultaneously with the work of Prof. Wülker the data on karyotype and chromosomal polymorphism of *Ch. heteropilicornis* from Siberian populations were published by Kiknadze et al. (1996). In this work, the species was described as *Chironomus* sp. *Ya2*, but later was identified as *Ch. heteropilicornis*. Some information on karyotype of *Ch. heteropilicornis* from Germany was presented by Kiknadze et al. (2016). In addition, Kiknadze et al. (2016) revised the mapping of *Ch. heteropilicornis* banding sequences in comparison with mappings of Wülker (1996) and Kiknadze et al. (2004).

The GenBank database does not contain any sequences of the COI gene of *Ch. heteropilicornis*. At the same time, in the BOLD database there are five sequences of the gene of *Ch. heteropilicornis* obtained from an imago collected from Trondheim region in Norway (Ratnasingham and Hebert 2007, accession numbers CHMNO266-15, CHMNO267-15, CHMNO268-15, CHMNO269-15, CHMNO413-15).

The aim of the work was to present the description of karyotype characteristics, chromosomal polymorphism and gene COI sequences of *Ch. heteropilicornis* from the South Caucasus. In addition, it was also very important to compare the chromosomal polymorphism characteristics and DNA data of *Ch. heteropilicornis* from the Caucasus with earlier studies.

## Methods

**Figure 1.**
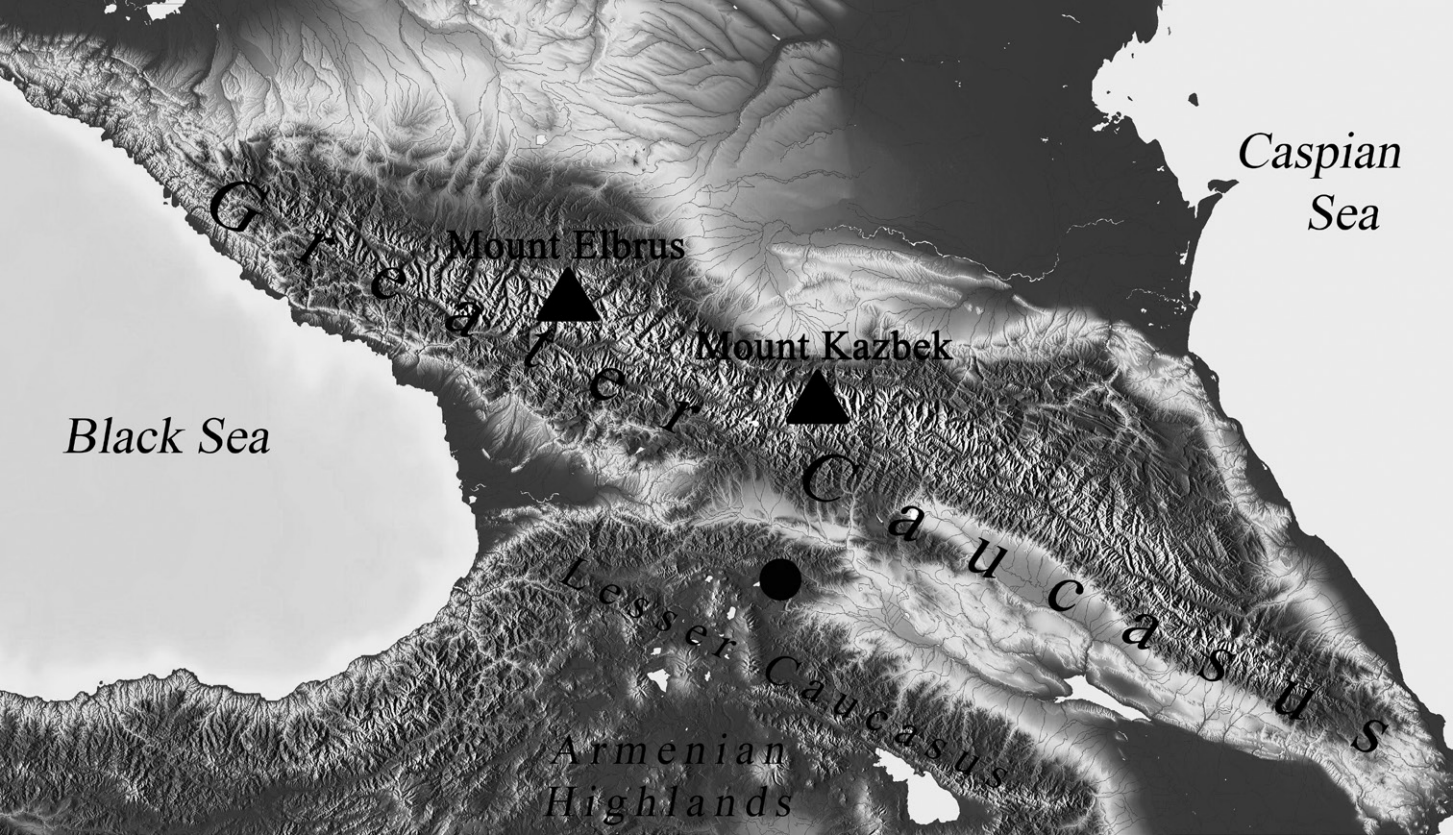
Collection site of *Ch. heteropilicornis* in South Caucasus. Collection site is marked with dark circle.

We used fourth instar larvae of *Ch. heteropilicornis* for both DNA and karyological study. We provide the collection sites and abbreviations of earlier studied populations (Kiknadze et al. 1996) in Table 1. We collected larvae from one site of the Republic of Georgia: 18.07.17, 41°38.936'N, 44°12.794'E, Tsalka district in the region of Kvemo Kartli, the lake situated 1.7 km east of Imera settlement, altitude ca 1600 m a.s.l. The lake has a circle shape, max. depth is about 1 m and water salinity is about 40 ppm. The collection site is marked on the map with a dark circle (Fig. 1). The geographic division of the Caucasus follows Gvozdetskii (1963). The area to the west of Mount Elbrus considered as the West Caucasus. The area between Mount Elbrus and Mount Kazbek considered as the Central Caucasus, and the area to the east of Mount Kazbek as the East Caucasus. The area that includes the Colchis Lowland, the Kura-Aras Lowland, the Lesser Caucasus, the Talysh Mountains, the Lenkoran Lowland and eastern portion of the Armenian Highlands is considered as the South Caucasus or Transcaucasia.

**Table 1.** Collection sites and number of analyzed *Ch. heteropilicornis* larvae from Siberian populations (Republic of Sakha (Yakutia)) per Kiknadze et al. (1996).

**Localities**	**Population abbreviation**	**Collection sites**	**Collection date**	**Number of specimens**
Siberian populations	VD-BA	Verkhnevilyuysky District, Khoro village, Bakyn lake	18.07.94	48
	NA-LA1	Nyurbinsky District, Antonovka village, lake without name	21.07.94	20
	NA-LA2	Nyurbinsky District, Antonovka village, lake for irrigation	21.07.94	22
	MM-UR	Mirninsky District, Mirny town, Irelyakh river	19.09.94	10

Thus, the collection site from the Republic of Georgia belongs to the South Caucasus or Transcaucasia. Regarding vertical zonation (Sokolov and Tembotov 1989), the site belongs to the Javakheti-Armenian variant.

The head capsule and body of 10 larvae were slide mounted in Fora-Berlese solution. The specimens have been deposited in the Tembotov Institute of Ecology of Mountain territories RAS in Nalchik, Russia. We studied the karyotype and chromosomal polymorphism in 33 larvae from the Caucasus region.

We fixed the larvae for karyological study in ethanol-glacial acetic acid solution (3:1). The slides of the chromosomes were prepared using the ethanol-orcein technique (see Dyomin and Iliynskaya 1988, Dyomin and Shobanov 1990). The banding sequences were designated per the accepted convention specifying the abbreviated name of the species, symbol of chromosome arm, and sequence number, as in hpiA1, hpiA2, etc. (Keyl 1962, Wülker and Klötzli 1973).

We performed the identification of chromosome banding sequences for arms A, E and F using the photomaps of Wülker (1996) and Kiknadze et al. (1996, 2016) in the system of Keyl (1962) and chromosome mapping for arms C and D as per Wülker (1996) and Kiknadze et al. (1996, 2016) in the system of Dévai et al. (1989).

We studied the chromosome slides using a Carl Zeiss Axio Imager A2 microscope and performed the statistical data processing using software package STATISTICA 10 (StatSoft).

We used the following parameters of chromosomal polymorphism characteristics for comparison: percentage of heterozygous larvae, average number of heterozygous inversions per larva. We calculated the genetic distances between populations according to Nei criteria (Nei 1972) using Chironomus 1.0 software (Kazakov and Karmokov 2015) based on original data along with Kiknadze et al. (1996).

### DNA extraction, amplification and sequencing

We used bodies of five larvae of *Ch. heteropilicornis* previously studied karyologically for further DNA extraction. DNA was extracted from the whole larva body using the Qiagen DNeasy Blood and Tissue Kit (Qiagen, Hilden, Germany) following the manufacturer’s protocol for animal tissue. DNA extraction was performed on vacuum-dried samples without prior homogenization. Samples were incubated in lysis buffer for 24 h. After extraction, the head capsules were retrieved for dry mounting. The barcoding region of the mitochondrial cytochrome oxidase subunit I (COI) gene was amplified using the Folmer et al. (1994) primers: LCO1490 (5'-GGTCAACAAATCATAAAGATATTGG-3') and HCO2198 (5'-TAAACTTCAGGGTGACCAAAAAATCA-3'). PCR was performed in a 25-µL reaction volume containing 2.5 mM MgCl2, 0.4 µg BSA, 0.8 mM GeneAmp dNTP Mix (Applied Biosystems), 0.5 µM of each primer, 1U of ABI AmpliTaq DNA Polymerase (Applied Biosystems, Foster City, CA, USA), 1X PCR buffer II (Applied Biosystems) and 3 µl template DNA extract.

The amplification profile consisted of an initial step of 94 °C for 2 min, followed by 30 cycles of 94 °C for 1 min, 50 °C for 30 s and 72 °C for 2 min, and finally a 10 min extension step at 72 °C. The PCR products were purified with Illustra ExoStar 1-Step (GE Healthcare).

Purified PCR products were sequenced (in both directions) externally by StarSeq GmbH (Mainz, Germany). The GenBank accession numbers of three sequences obtained in this study (South Caucasus) are provided in Table 2.

**Table 2.** Collection sites and accession numbers of *Ch. pilicornis* and *Ch. heteropilicornis* nucleotide sequences used in the study.

**Species**	**GenBank and BOLD accession number**	**Localities**
*Ch. pilicornis*	CNQUF171-14	Canada
	INNV033-08	Canada
	ARCHR033-11	Greenland
	ARCHR026-11	Greenland
	BSCHI735-17	Sweden
	BSCHI736-17	Sweden
*Ch. heteropilicornis*	CHMNO266-15	Norway
	CHMNO269-15	Norway
	CHMNO268-15	Norway
	CHMNO267-15	Norway
	CHMNO413-15	Norway
	MK795770	South Caucasus
	MK795771	South Caucasus
	MK795772	South Caucasus

### Phylogenetic analysis

For the phylogenetic comparison we used DNA data from both GenBank and BOLD databases for the species *Ch. balatonicus* Devai et al., 1983 (JN016826.1, AF192193.1), *Ch. plumosus* (Linnaeus, 1758) (KF278218.1, KF278217.1), *Ch. usenicus* Loginova & Beljanina, 1994 (JN016817.1, JN016806.1), *Ch. entis* Shobanov, 1989 (KM571024.1), *Ch. borokensis* (Kerkis et al., 1988) (AB74026.1), *Ch. muratensis* Ryser, Scholl & Wuelker, 1983 (AF192194.1), *Ch. curabilis* Belyanina, Sigareva & Loginova, 1990 (JN016822.1, JN016810.1), *Ch. nuditarsis* Str. (Keyl, 1961) (KY225345.1), *Ch. dorsalis* Meigen, 1818 (KY838605.1), *Ch. salinarius* Kieffer, 1915 (KR641621.1), *Ch. tentans* (Fabricius), 1805 (AF110157.1), *Ch. pallidivitattus* sensu Edwards, 1929 (AF110165.1), *Ch. dilutus* Shobanov et al., 1999 (JF867805.1), *Ch. nipponensis* Tokunaga, 1940 (LC096172.1), *Ch. cingulatus* Meigen, 1830 (AF192191.1), *Ch.* “annularius” sensu Strenzke (1959) (AF192189.1), *Ch. bernensis* Klotzli, 1973 (AF192188.1), *Ch. commutatus* Keyl, 1960 (AF192187.1), *Ch. novosibiricus* Kiknadze et al., 1993 (AF192197.1), *Ch. tuvanicus* Kiknadze et al., 1993 (AF192196.1), *Ch. whitseli* Sublette & Sublette, 1974 (KR683438.1), *Ch. maturus* Johannsen, 1908 (DQ648204.1), *Ch. acutiventris* Wulker, Ryser & Scroll, 1983 (AF192200.1), *Ch. heterodentatus* Konstantinov, 1956 (AF192199.1), *Ch. melanescens* Keyl, 1961 (MG145351.1), *Ch. aprilinus* Meigen, 1818 (KC250746.1), *Ch. luridus* Strenzke, 1959 (AF192203.1), *Ch. pseudothummi* Strenzke, 1959 (KC250754.1), *Ch. riparius* Meigen, 1804 (KR56187.1), *Ch. piger* Strenzke, 1959 (AF192202.1) and *Drosophila melanogaster* (Meigen, 1830) (BBDEE689-10).

We provide our DNA data for *Ch. pilicornis* and *Ch. heteropilicornis* with corresponding accession numbers and collection sites in Table 2. We conducted the alignment of COI nucleotide sequences by MUSCLE with a genetic code of “invertebrate mitochondrial” packaged in MEGA 6 (Tamura et al. 2013). We calculated the pairwise sequence distances (Table 6) consisting of the estimated number of base substitutions per site using MEGA 6 and the K2P model (Kimura 1980). The analysis involved 13 nucleotide sequences. Codon positions included were 1^st^+2^nd^+3^rd^+Noncoding. All positions containing gaps and missing data were eliminated. There were in total 614 positions in the final dataset.

We conducted the estimation of phylogenetic relationships by the Bayes algorithm implemented in MrBayes 3.2.6 (Ronquist and Huelsenbeck 2003) for 1,000,000 iterations and 1000 iterations of burn in. We used the GTR with gamma distribution and invariant sites (GTR+I+G) model. We performed the determination of the appropriate model in MEGA 6 (Tamura et al. 2013). Our analysis involved 49 nucleotide sequences. We eliminated all positions with less than 95% site coverage. There were 579 positions in the final dataset. The COI sequence of *Drosophila melanogaster* (BOLD accession number BBDEE689-10) was used as outgroup.

## Results

We identified the larvae belonging to the genus *Chironomus* Meigen, 1803 in the studied site as *Ch. heteropilicornis* by both morphological and chromosomal characteristics. The morphological larval characteristics of *Ch. heteropilicornis* from the Caucasian site are similar to those previously described for this species by Wülker (1996) and Kiknadze et al. (1996).

### Karyotype of *Ch. heteropilicornis* from the South Caucasus.

**Figure 2.**
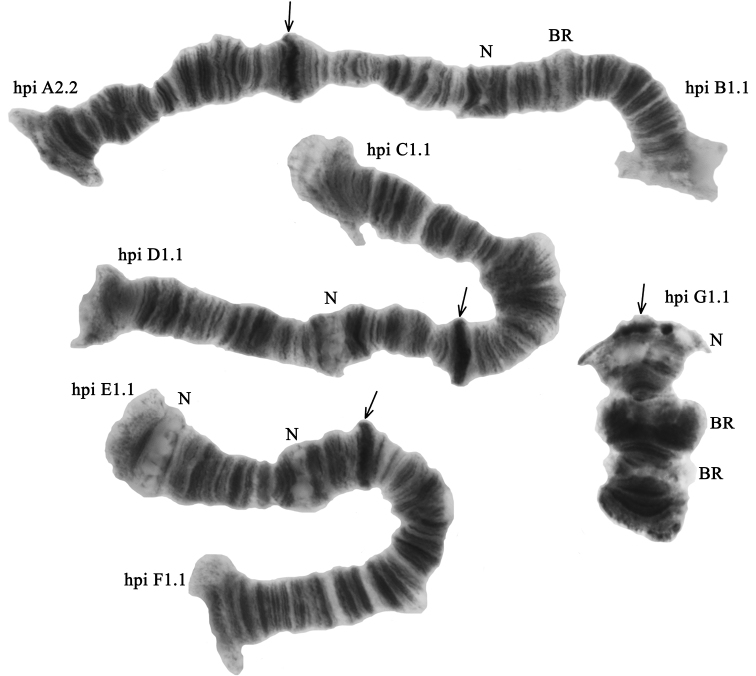
Karyotype of *Ch. heteropilicornis* from the South Caucasus; hpiA2.2, hpiD1.1 etc. – genotypic combinations of banding sequences; BR – Balbiani rings, N – nucleolus. Arrows indicate centromeric bands.

The diploid number of chromosomes in *Ch. heteropilicornis* karyotype is 2n = 8, chromosome arm combination is AB, CD, EF, and G (the “thummi” cytocomplex) (Fig. 2). Chromosomes AB and CD are metacentric, EF is submetacentric, and G is telocentric. There are five permanent nucleoli (N) in the karyotype: arms B, D and G contain one nucleolus, arm E has two. There are three Balbiani rings (BR) in the karyotype: two in the arm G and one in the arm B (Fig. 2). The homologues in the arm G lie close to each other or are tightly paired. The centromeric bands are prominent and heterochromatic.

### Banding sequences and chromosomal polymorphism of *Ch. heteropilicornis* from the South Caucasus.

Previously, Kiknadze et al. (2016) described 15 banding sequences in *Ch. heteropilicornis* banding sequences pool. In the studied Caucasian population, seven of those sequences are present, and one banding sequence has been found for the first time, providing in total eight banding sequences in the population from Caucasus (Table 3).

**Figure 3.**
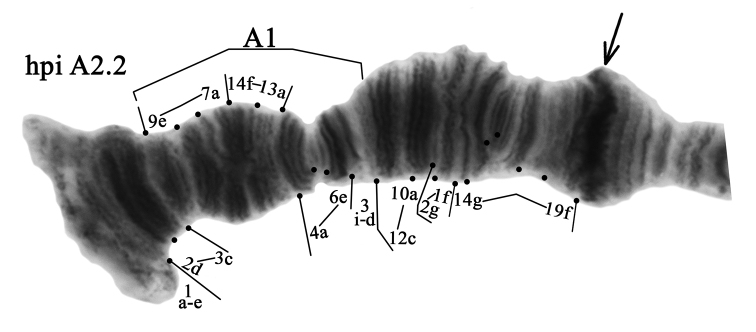
Homozygous genotypic combination hpiA2.2. Designations as in Fig. 2.

**Arm A** has one banding sequence, which we designated as hpiA2 (Figs 2, 3, Table 3). The banding sequence hpiA2 is new for the species and described for the first time (Fig. 3, Tables 3, 4). It differs from hpiA1 by one simple inversion step that involves regions **3d-i 6e-4a 13a-14f 7a-9e**:

**Table 3.** Frequencies of banding sequences in different populations of *Ch. heteropilicornis*. N – the number of individuals, * – original data.

**Banding sequences**	**Eastern Siberia (Yakutia)**				**South Caucasus**
	**VD-BA**	**NA-LA1**	**NA-LA2**	**MM-IR**	**SC-SJ**
	**N=48**	**N=20**	**N=32**	**N=10**	N=33*
A1	1.000	1.000	0.977	1.000	0
AX	0	0	0.023	0	0
A2	0	0	0	0	1.000
B1	1.000	1.000	1.000	1.000	1.000
C1	0.677	0.800	0.796	0.700	1.000
C2	0.313	0.175	0.204	0.300	0
C3	0.010	0.025	0	0	0
D1	0.167	0	0.068	0.150	1.000
D2	0.833	1.000	0.932	0.850	0
E1	1.000	1.000	1.000	1.000	1.000
F1	0.740	0.775	0.864	0.750	0.955
F2	0.042	0	0.046	0.100	0.045
F3	0.218	0.225	0.090	0.150	0
G1	1.000	1.000	1.000	1.000	1.000

hpiA2 1a-e 2d-3c **9e-7a 14f-13a 4a-6e 3i-d** 12c-10a 2g-1f 14g-19f C

According to Kiknadze et al. (1996, 2016) in the populations from Yakutia, two banding sequences were present in the arm A: the standard hpiA1 and an inverted one designated by authors as hpiA2. Unfortunately, the latter was not mapped and the chromosome slide containing this banding sequence, as well as its photos, were not preserved (personal communication of Veronika V. Golygina). Due to this, the banding sequence found in Siberia cannot be compared with banding sequence found in Caucasian population, as well as with any other banding sequence that may be found in populations of *Ch. heteropilicornis* in the future. Therefore, we propose to designate Caucasian sequence as hpiA2 and Siberian one as hpiAX.

**Arm B** was monomorphic with banding sequence hpiB1.1 (Fig. 2, Tables 3, 4).

**Figure 4.**
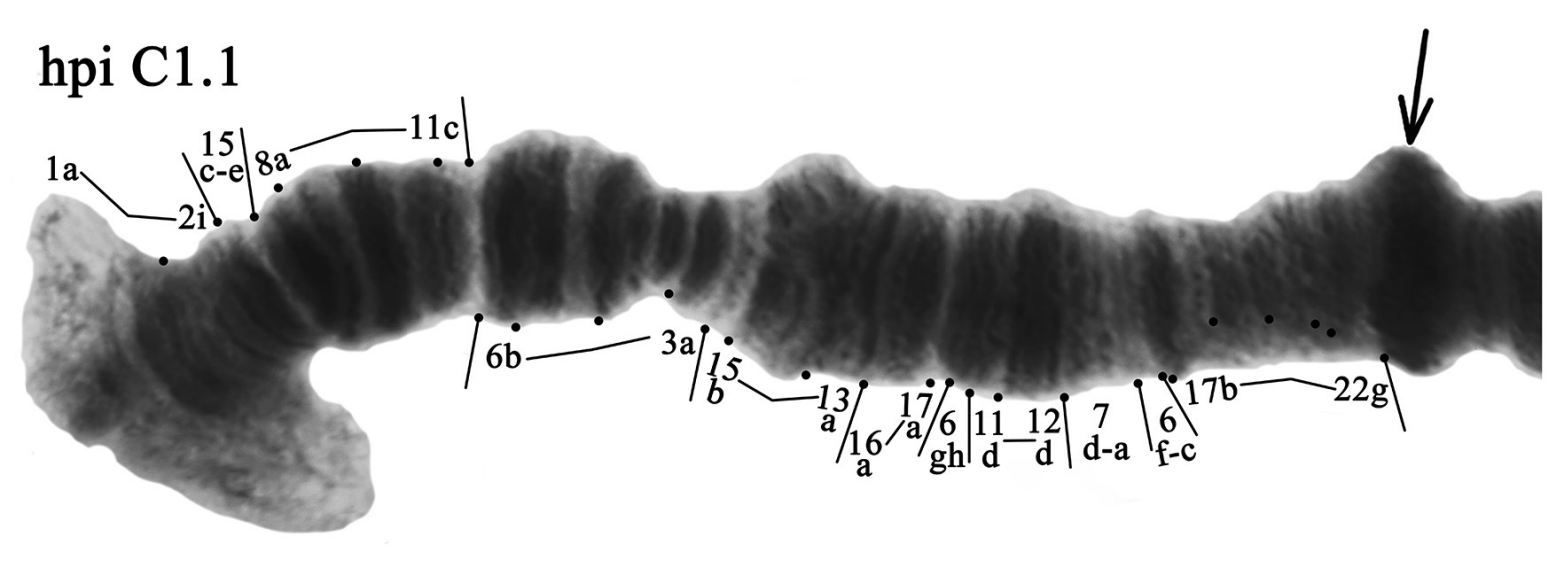
Homozygous genotypic combination hpiC1.1. Designations are as in Fig. 2.

**Arm C** was monomorphic with banding sequence hpiC1.1 (Fig. 4, Tables 3, 4).

**Figure 5.**
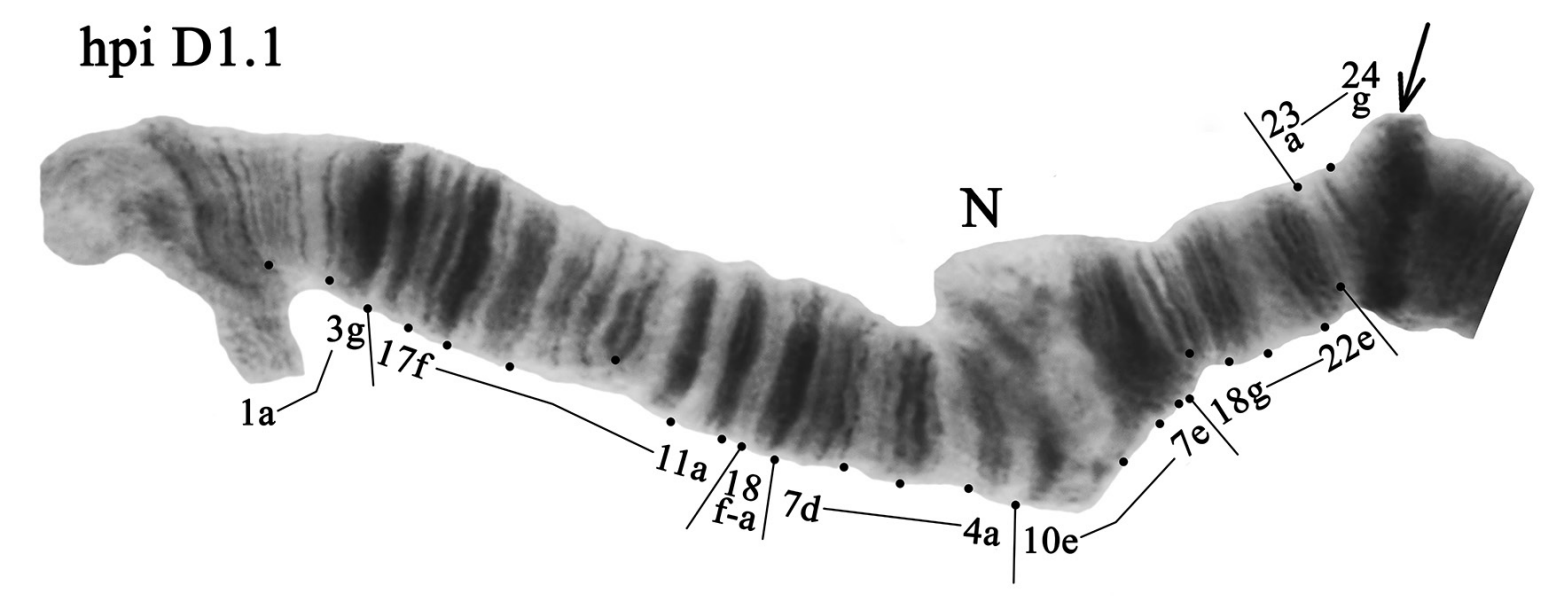
Homozygous genotypic combination hpiD1.1. Designations are as in Fig. 2.

**Arm D** also was monomorphic with banding sequence hpiD1.1 (Fig. 5, Tables 3, 4).

**Figure 6.**
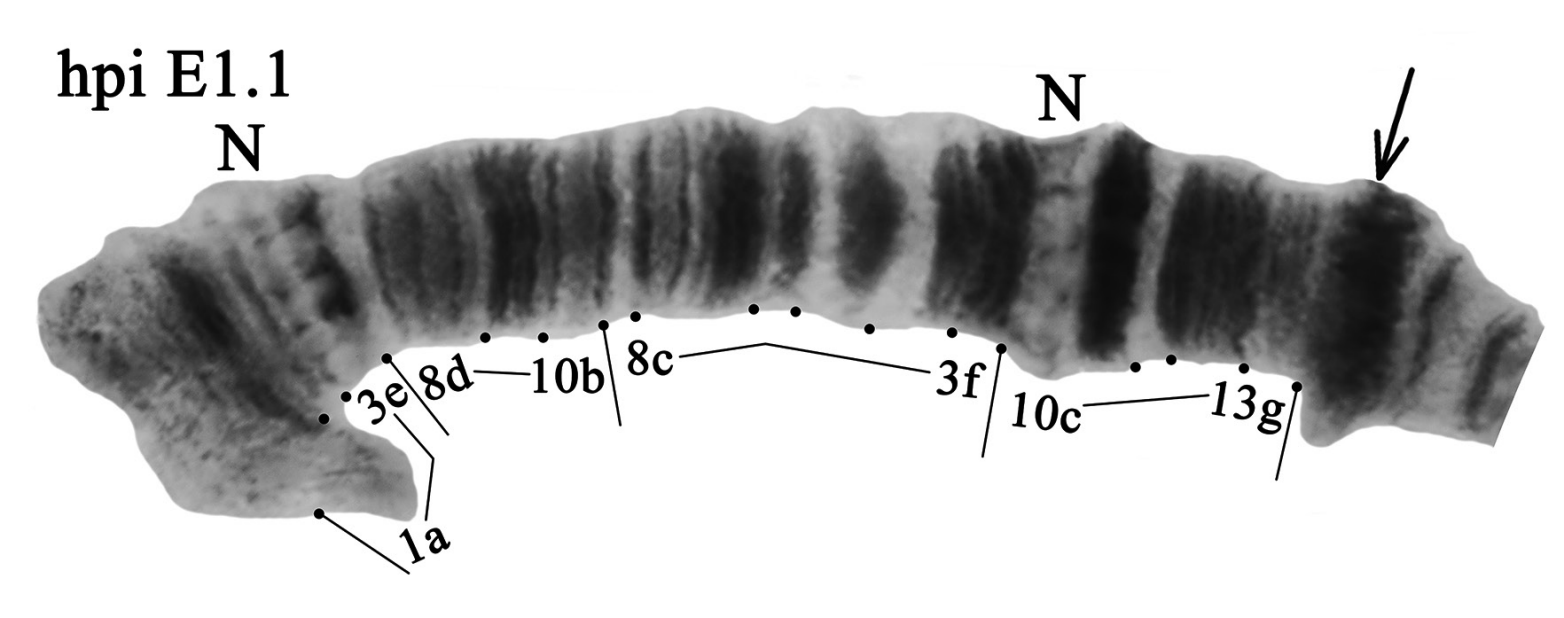
Homozygous genotypic combination hpiE1.1. Designations are as in Fig. 2.

**Arm E** was monomorphic with banding sequence hpiE1.1 (Fig. 6, Tables 3, 4). We found that banding patterns in the arm E from Caucasian population are the same as in the photos from German and Siberian populations (Kiknadze, Istomina 2011; Kiknadze et al. 2016), but in our opinion the photos from the Caucasus has slightly better banding structure. Based on analysis of these new photos we suggest that the mapping of the arm should be revised.

**Table 4.** Frequencies of genotypic combinations in different populations of *Ch. heteropilicornis*. N – the number of individuals, * – original data.

**Genotypic combinations**	**Eastern Siberia (Yakutia)**				**South Caucasus**
	**VD-BA**	**NA-LA1**	**NA-LA2**	**MM-IR**	**SC-SJ**
	**N=48**	**N=20**	**N=32**	**N=10**	N=33*
A1.1	1.000	1.000	0.955	1.000	0
A1.X	0	0	0.045	0	0
A2.2	0	0	0	0	1.000
B1.1	1.000	1.000	1.000	1.000	1.000
C1.1	0.438	0.600	0.636	0.500	1.000
C1.2	0.458	0.350	0.318	0.400	0
C2.2	0.083	0	0.046	0.100	0
C1.3	0.021	0.050	0	0	0
D1.1	0	0	0	0	1.000
D1.2	0.333	0	0.137	0.300	0
D2.2	0.667	1.000	0.863	0.700	0
E1.1	1.000	1.000	1.000	1.000	1.000
F1.1	0.500	0.550	0.728	0.500	0.909
F1.2	0.042	0	0.090	0.200	0.091
F2.2	0.021	0	0	0	0
F1.3	0.438	0.450	0.182	0.300	0
G1.1	1.000	1.000	1.000	1.000	1.000
Heterozygous larvae, %	85	75	55	70	9
Average number of heterozygous inversions per larvae	1.3	0.9	0.7	1.2	0.09

The previous mapping of the arm as per Wülker (1996) and Kiknadze et al. (1996, 2016) was as follows:

hpiE1.1 1a-3e 10b-3f 10c-13g C

We propose a slightly different version of mapping:

hpiE1.1 1a-3e **8d-10d** 8c-3f 10c-13g C

In the new photos, one can clearly see that there is an inversion in the region 10b-8d.

**Figure 7.**
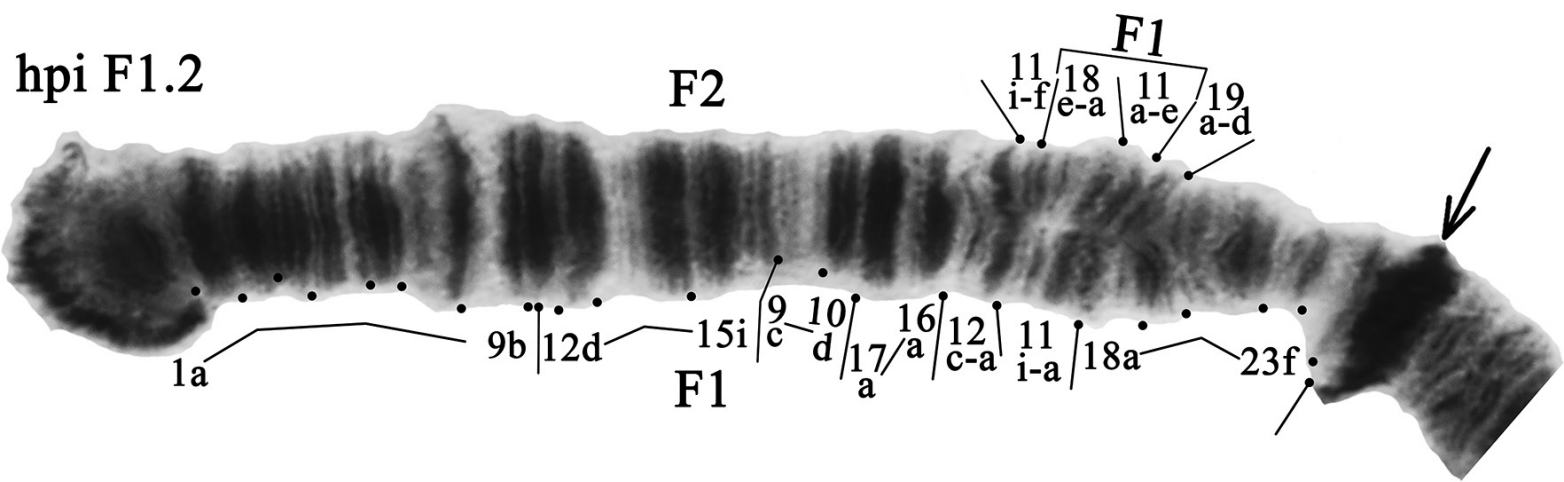
Heterozygous genotypic combination hpiF1.2. Designations as in Fig. 2.

**Arm F** has two banding sequences: hpiF1 and hpiF2 (Fig. 7). The banding sequence hpiF1 and genotypic combination hpiF1.1 were predominant in the population of South Caucasus (Tables 3, 4). The sequence hpiF2 has been observed only in the heterozygote state (Table 4) and with a rather low frequency (0.091). As with the arm E, the new photos of the arm F from Caucasian population have slightly better banding structure and, as the banding pattern in the arm is the same as in German and Siberian populations (Kiknadze, Istomina 2011), we were able to suggest some correction for mapping of hpiF2.

The previous mapping of the arm as per Kiknadze et al. (2016) was as follows:

hpiF2 1a-9b 12d-15i 9c-10d 17d-16a 12c-a 11ih 19d-18a 11a-g 20a-23f

We propose the new version of mapping:

hpiF2 1a-9b 12d-15i 9c-10d 17d-16a 12c-a 11i-f **18e-a 11a-e** 19a-23f

Our mapping shows that the inversion step that differ sequences hpiF1 and hpiF2 was slightly larger than described by Kiknadze et al. (1996), but smaller than described by Kiknadze et al. (2016).

**Arm G** was monomorphic with banding sequence hpiG1.1 (Fig. 2, Tables 3, 4).

We compared the chromosomal polymorphism of *Ch. heteropilicornis* from the Caucasian population with that of populations from other regions.

Wülker (1996) described nine banding sequences in the populations of Finland and Sweden: hpiA1, hpiB1, hpiC1, hpiC2, hpiD1, hpiE1, hpiF1 (after revision designated as hpiF3), hpiF2 (after revision designated as hpiF1) and hpiG1. Unfortunately, he did not provide the data on frequencies of the banding sequences and genotypic combinations so it was impossible to compare quantitative data.

The data for Siberian populations are available due to Kiknadze et al. (1996).

**Arm A.** In the populations of Finland and Sweden (Wülker 1996) only one banding sequence – hpiA1 – has been observed. Most of Siberian populations (Kiknadze et al. 1996) are also characterized by the presence of the one banding sequence, hpiA1. Only one population contained another banding sequence – hpiAX – in the heterozygote state and with very low frequency (Table 3). In the populations of South Caucasus, only one banding sequence – hpiA2 – has been observed. As it was dominant in Caucasus population and completely absent elsewhere, we believe that it might be endemic to this region (Table 3).

**Arm B** has been monomorphic in all populations studied up to date, including the population of South Caucasus (Tables 3, 4).

**Arm C.** In the populations of Finland and Sweden (Wülker 1996) two banding sequence – hpiC1 and hpiC2 – has been observed. The arm also was polymorphic in all the Siberian populations. Out of three banding sequences found in the arm, two – hpiC1 and hpiC2 – were observed both in homo- and heterozygote state with hpiC1 being predominant in all populations, and hpiC3 was found in heterozygote state only (Tables 3, 4). However, the arm was monomorphic in population from South Caucasus with only one genotypic combination – hpiC1.1 – present.

**Arm D.** In the populations of Finland and Sweden (Wülker 1996) only one banding sequence – hpiD1 – has been observed. The arm was polymorphic in most of the Siberian populations (Table 3). Two banding sequences – hpiD1 and hpiD2 – were found with the former observed in heterozygous state only. The genotypic combination hpiD2.2 was predominant in most of the Siberian population (Table 4) but completely absent in population from South Caucasus where only genotypic combination hpiD1.1 was found.

**Arm E** has been monomorphic in all studied populations (Tables 3, 4).

**Arm F.** In the populations of Finland and Sweden (Wülker 1996) two banding sequence – hpiF1 and hpiF3 – has been observed. The arm was also polymorphic both in all Siberian and in South Caucasus populations (Tables 3, 4). Three banding sequences were present in the arm in most of the Siberian populations but only two were found in population from South Caucasus. Banding sequences hpiF1 and hpiF2 were found in both Siberian and South Caucasus populations with hpiF1 predominant in all of them (Table 4). Banding sequence hpiF2 was found mostly as heterozygous combination hpiF1.2 in all populations of Siberia but in one population from Siberia homozygote hpiF2.2 has been observed. Banding sequence hpiF3 was present in populations from Siberia and absent in Caucasian population. It is interesting to note that it showed rather high frequencies yet was found only as heterozygotes with hpiF1.

**Arm G** was monomorphic in all the studied populations with banding sequence hpiG1 and genotypic combination hpiG1.1 (Tables 3, 4).

The level of inversion polymorphism in Caucasian *Ch. heteropilicornis* population is quite low in comparison with previously studied populations (Table 4). The percentage of heterozygous larvae in the population of the South Caucasus is low (9%), while in the Siberian populations this percentage is much higher and varies from 55% to 85% (Table 4). The average number of heterozygous inversions per larvae is also very low (0.09), while in the Siberian populations this number varies from 0.7 to 1.3 (Table 4).

Kiknadze et al. (1996) found the heterozygosity of centromeric band’s size in one site (MM-UR) of Siberia (Table 1). The larvae with heterozygosity of thick and thin centromeric bands in the AB and EF chromosomes were observed. We did not find such type of the chromosomal polymorphism in population of South Caucasus.

According to Kiknadze and Istomina (2011), Siberian and European (Germany) populations differed by very high frequency of the homozygotes hpiD2.2 in Siberia; the sequence hpiD1 were found only as heterozygote hpiD1.2 in Siberia, while it was dominated in Europe as hpiD1.1.

**Figure 8.**
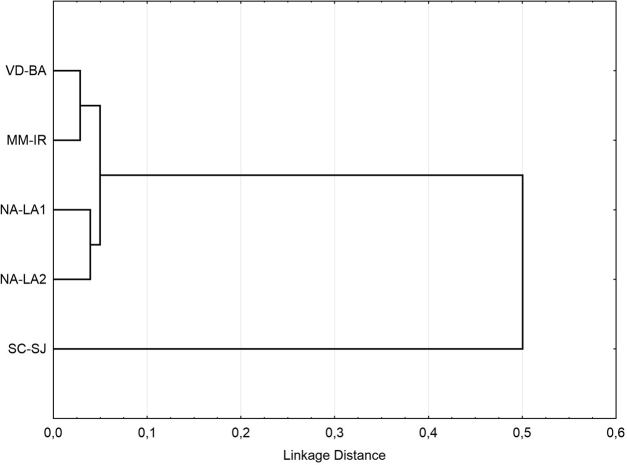
Tree dendrogram for five *Ch. heteropilicornis* populations, *single linkage, Euclidean distances.* For abbreviations of the populations, see Table 1.

In the dendrogram of genetic distances (Fig. 8), calculated on the basis of frequencies of genotypic combinations in different populations (Table 4) using Nei criteria (Nei 1972), Siberian populations form one clear cluster. The population from the South Caucasus (SC-SJ) does not belong to this cluster. The distance (Table 5) between populations of Siberia (0.005–0.023) is lower than the values that characterize different population of one species (0.136 ± 0.026, Gunderina 2001), so Siberian populations could be considered as truly belonging to one big population. At the same time, the distance between populations of Siberia and the population of South Caucasus (0.379–0.445) is higher than the distance between different population of the same species and almost reach the mean distance (0.474 ± 0.314) between subspecies (Gunderina 2001). Due to this, we can assume that the population of the South Caucasus separated from Siberian populations at the level of subspecies.

**Table 5.** Values of genetic distances between different populations of *Ch. heteropilicornis* calculated using Nei criteria (Nei 1972).

**Population**	**VD-BA**	**NA-LA1**	**NA-LA2**	**MM-IR**	**SC-SJ**
VD-BA	0				
NA-LA1	0.023	0			
NA-LA2	0.023	0.014	0		
MM-IR	0.005	0.023	0.014	0	
SC-SJ	0.444	0.445	0.376	0.423	0

Overall, we successfully obtained three sequences of *Ch. heteropilicornis* from five Caucasian larvae (Table 2). All three sequences had the same haplotype.

Calculated pairwise sequence distances (Table 6) consisting of the estimated number of base substitutions per site using K2P model (Kimura 1980) show an interesting picture. The distances between Norwegian sequences of *Ch. heteropilicornis* are pretty low and varies from 0% to 0.2%. The distance value between sequences of *Ch. heteropilicornis* of Caucasus is 0 as they all have the same haplotype. The distances between sequences of *Ch. heteropilicornis* from Norway and sequences of *Ch. heteropilicornis* from Caucasus varies from 2.0% to 2.2%. The distances between sequences of *Ch. heteropilicornis* from Norway and *Ch. pilicornis* sequences from Canada-Greenland varies from 5.1% to 5.3%. The distance between sequences of *Ch. heteropilicornis* from Caucasus and *Ch. pilicornis* sequences from Canada-Greenland is the largest among compared populations and reaches 5.6%. The distance between sequences of *Ch. heteropilicornis* from Caucasus and sequences of *Ch. pilicornis* from Sweden reaches 2%. Surprisingly, the distance between sequences of *Ch. heteropilicornis* from Norway and *Ch. pilicornis* from Sweden is pretty low and varies from 0 to 0.2%, that is quite similar to the picture observed in the Norwegian population. The sequences of *Ch. heteropilicornis* from Norway CHMNO267-15, CHMNO268-15, CHMNO413-15 and *Ch. pilicornis* sequences from Sweden BSCHI735-17, BSCHI736-17 have the same haplotype. Finally, the distance between sequences of *Ch. pilicornis* from Canada-Greenland and sequences of *Ch. pilicornis* from Sweden reaches 5.1%.

**Table 6.** Estimates of evolutionary divergence between sequences of *Ch. heteropilicornis* and *Ch. pilicornis*. The number of base substitutions per site (%) from between sequences are shown. Analyses were conducted using the Kimura 2-parameter model (Kimura 1980).

	***Ch. heteropilicornis*, Norway**					***Ch. heteropilicornis*, Caucasus**			***Ch. pilicornis***				
	**1**	**2**	**3**	**4**	**5**	**1**	**2**	**3**	**Sweden 1**	**Sweden 2**	**Greenland 1**	**Greenland 2**	**Canada**
CHMNO266-15*Ch. heteropilicornis* Norway_1													
CHMNO267-15*Ch. heteropilicornis* Norway_2	0.2												
CHMNO268-15*Ch. heteropilicornis* Norway_3	0.2	0.0											
CHMNO269-15*Ch. heteropilicornis* Norway_4	0.0	0.2	0.2										
CHMNO413-15*Ch. heteropilicornis* Norway_5	0.2	0.0	0.0	0.2									
MK795770*Ch. heteropilicornis* Caucasus_1	2.2	2.0	2.0	2.2	2.0								
MK795771*Ch. heteropilicornis* Caucasus_2	2.2	2.0	2.0	2.2	2.0	0.0							
MK795772*Ch. heteropilicornis* Caucasus_3	2.2	2.0	2.0	2.2	2.0	0.0	0.0						
BSCHI736-17*Ch. pilicornis* Sweden_1	0.2	0.0	0.0	0.2	0.0	2.0	2.0	2.0					
BSCHI735-17*Ch. pilicornis* Sweden_2	0.2	0.0	0.0	0.2	0.0	2.0	2.0	2.0	0.0				
ARCHR033-11*Ch. pilicornis* Greenland_1	5.3	5.1	5.1	5.3	5.1	5.6	5.6	5.6	5.1	5.1			
ARCHR026-11*Ch. pilicornis* Greenland_2	5.3	5.1	5.1	5.3	5.1	5.6	5.6	5.6	5.1	5.1	0.0		
INNV033-08 *Ch. pilicornis* Canada	5.3	5.1	5.1	5.3	5.1	5.6	5.6	5.6	5.1	5.1	0.0	0.0	

**Figure 9.**
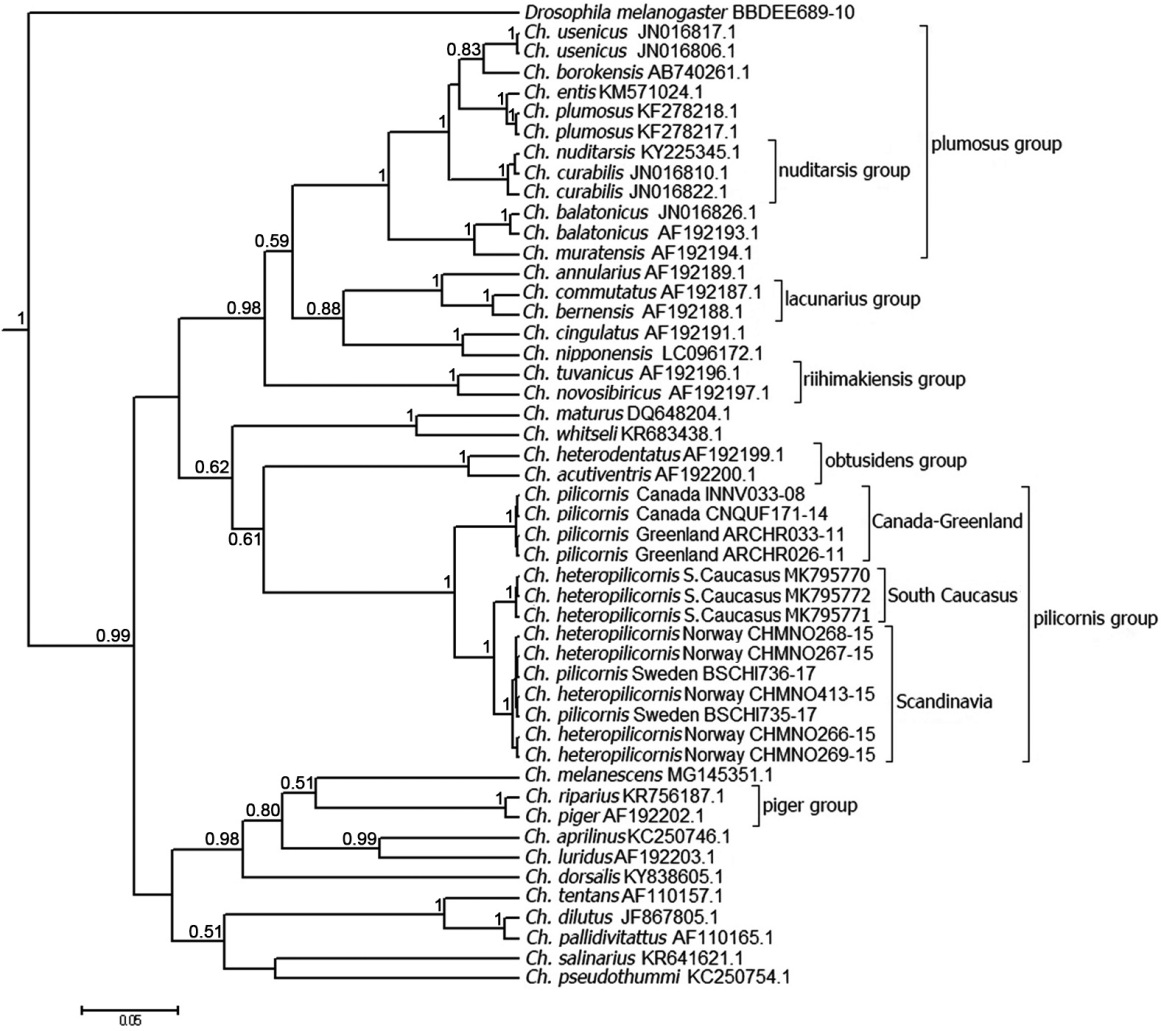
Phylogenetic tree of *Chironomus* species estimated by the Bayesian inference (BA). Support values are given if they exceed 0.5. The numbers at the nodes indicate posterior probabilities.

In the phylogenetic tree of *Chironomus* species, constructed with method of the Bayesian inference (Fig. 9), we can see several clear clusters with rather high probabilities that correspond to the groups of closely related species, such as *Ch. plumosus* group, *Ch. nuditarsis* group, *Ch. riihimakiensis* group, *Ch. lacunarius* group, *Ch. obtusidens* group, *Ch. piger* group and *Ch. pilicornis* group. Predictably, the sequences of *Ch. heteropilicornis* and *Ch. pilicornis* form clear separate cluster with high support value that corresponds to *Ch. pilicornis* group. At the same time, one can see an interesting picture inside this cluster. There is a separate branch of *Ch. pilicornis* sequences from Canada and Greenland. There is another larger branch of *Ch. heteropilicornis* sequences. Inside this branch, there are two separate lines and the first one is the branch of Caucasian *Ch. heteropilicornis* sequences. The second branch consists of *Ch. heteropilicornis* sequences from Norway and, surprisingly, of *Ch. pilicornis* sequences from Sweden.

## Discussion

We found the species *Ch. heteropilicornis* in the South Caucasus for the first time.

Overall, we can characterize the Caucasian population of the species as having a low level of polymorphism. We found one new banding sequence hpiA2 in the banding sequences pool of *Ch. heteropilicornis*. We observed inversion polymorphism only in the arm F.

The dendrogram of genetic distances (Fig. 8) by Nei criteria (Nei 1972), calculated using karyological data, shows a clear separation of the Caucasian population from populations of Siberia. At the same time, the distance between populations of Siberia and the population of South Caucasus (0.379–0.445) almost reach the mean value (0.474 ± 0.314) for the subspecies (Gunderina 2001). Due to this, we can assume that the population of South Caucasus separated from Siberian populations at the level of subspecies.

In the work of Proulx et al. (2013), where genetic, morphological and karyological observations were used to discriminate species of *Chironomus* from Canada, it was shown that intraspecific K2P distance for *Chironomus* species characterized by the COI gene range from zero to 3%. As was noted in that research, these values can be used as a reference in distinguishing *Chironomus* species using this approach, but data on COI gene should not be used without other methods.

Following Proulx et al. (2013) we can conclude that the genetic distances between *Ch. heteropilicornis* sequences from Norway and *Ch. heteropilicornis* sequences from the Caucasus (2.0–2.2%) are lower than the 3% interspecific threshold for genus *Chironomus*. As expected, the genetic distances between *Ch. heteropilicornis* and *Ch. pilicornis* sequences (5.1–5.6%) exceed the 3% range and correspond to separate species. However, the distance values between sequences of *Ch. heteropilicornis* from Norway-Caucasus and *Ch. pilicornis* sequences (BSCHI735-17, BSCHI736-17) from Sweden are lower (0–2.0%) than the interspecific threshold. Moreover, most of *Ch. heteropilicornis* sequences from Norway have the same haplotype as both *Ch. pilicornis* sequences from Sweden. Quite similar picture was observed in the groups of sibling species, such as the *Ch. plumosus* group, and *C. tentans* group (Martin et al. 2002, Guryev and Blinov 2002, Polukonova et al. 2009). If, in this particular case, it is not an error of species identification, which can happen quite often when only morphological methods are used, one of the possible explanations for this picture may be the same as was earlier proposed by Polukonova et al. (2009). It can be a result of interspecific hybridization with subsequent recurrent crosses resulting in the appearance of mtDNA of one of the parental species in the offsprings. In this case, even an insignificant selective advantage of this mtDNA is able to lead to a rapid fixation of the new haplotype in the population (Powell 1983, Guryev and Blinov 2002). Probably there was an interspecific hybridization event between *Ch. heteropilicornis* (female) and *Ch. pilicornis* (male) in population of Sweden. We suppose that it is quite possible because according to Wülker (1996) both species occurred sympatrically in collection site Kyrkösjärvi, Seinajöki-area (South Ostrobothnia, western Finland). The *heteropilicornis*-like sequences of *Ch. pilicornis* according to BOLD database were obtained from imago collected from Uppland on the eastern coast of Sweden, just north of Stockholm. This collection site is in 450 km south-west of collection site in western Finland, where both species occurred sympatrically. Probably such kind of hybridization events could have occurred more than once. To obtain a clearer picture it would be necessary to conduct simultaneous sequencing of genes of both mitochondrial and nuclear genomes of the same individuals with a preliminary cytogenetic analysis of both species from other sites of Sweden and Scandinavia.

All the obtained data indicate that the studied Caucasian population of *Ch. heteropilicornis* is a separate diverged population of the species on karyological and molecular-biological level. At the same time, the degree of this divergence by DNA data is lower than 3.0% threshold for *Chironomus* species.
